# Supplementary material for: Chromothripsis during telomere crisis is independent of NHEJ, and consistent with a replicative origin
Source: Genome Res. 2019 May;29(5):737–49. doi: 10.1101/gr.240705.118 (PMC6499312; doi:10.1101/gr.240705.118)
Supplement: Supplemental Material [file supp_gr.240705.118_Supplemental_file_1.zip › contigs/annotated_contigs/DB50/contig.2.DB50_length_842_mean_cov_5.52494061758.docx]

**DB50_length_842_mean_cov_5.52494061758**

```
AGAGCAAGTGTGTGTAGTGGGCCCCAGGCACTCACCATCTGCCATGCTCCCTTGTTCAAACCTCAACTATGGTCCTCAAAACCACATCA
 >chr20:15220221-15220608 - E=6e-218
CCATTGAGAGGGTAGAGATGGTAAAGAGAGCATTCAAACACAGCATAACCAGAAAATGAGGTTCTTCCACACATTTGTCACCTAGTCTA

AGTGTGTTAACCTATTTCTTTCTTTCTCCTGGCTTCTTTCTCCTTGAGTCAATGTGAGTCATGCCTCTTAATTTGGTCTATAAGCTTTT

CTGTTGTGCATCCTCCGGGATAGCTTACCTGCTAGTATACTGGGAGCAGGGAAATTTTCAGAACATGGGCTCAGAAGAGATGGAGAGAC

TGCACTAAAGTTACTCGTATGGGCTGAAATC|CATTTTCAGTCACAATTTAAATGTCGTGGTAATGGTTACTCCCAGAGGCTTCCAGTT
                                 >chr20:15125896-15126352 - E=1e-245
TCACATCCCAACTTGTTTCTTTTCTTAGTCTTTATTTGTACTGTACTAGAGAAGCCCTATTTAAAATTAATTGTCATTAAGAACAGTGG

CAAATGTTTGGTATTTCTTTCAAATATTTAGATGTCTTTAGAATAAAATGTTTCTGCCCAGAAAAGAAGGGTTCAGATTATTCAGACAT

CAAATACATTGCTGAGGGTGTCACTTGAATAACTGATACAAATAAAAGTGGCCTTTATTTGTATTATTGGTGTCGTATGAAAAACATGC

CTTTTTCAAAAAAAATAGAGTAATTCCTTTTTTCTCTACTCCCTGATGAACTTCAGTTTGGAAAAGCAGAAATTTATCCTTGAAACCCA

GAAAATAAATCCCAAAGACTTACACCTATGGCTATAATTTTT
```
